# Medical Opinion and Movement

**Published:** 1907-11-23

**Authors:** 


### MEDICAL OPINION AND MOVEMENT.

ONE of the problems that will have to be considered and faced under the "medical inspection of the children of the elementary school" is the care of the teeth. The report of Dr. Kerr, to which we have already referred, shows the deplorable condition of the mouths of the greater proportion of these children. The teaching of dental hygiene in the schools may do something in the right direction, but some provision for remedial treatment appears to be absolutely necessary. It is estimated that the present hospital accommodation in London would not suffice for more than 200 cases weekly, or 10,000 annually, and at least ten times this accommodation is necessary in order to relieve any considerable proportion of the children requiring treatment. When one considers the amount of permanent ill-health and defective development that results from dental caries, the importance of this aspect of school hygiene is overwhelming. There is probably no expenditure of public money which would return so great a gain in increased working efficiency of the community as the small expense that would be incurred by adequate provision for school dentistry. In Germany this fact has already been fully grasped. Some few years back a scheme was worked out by Professor Jessen for the municipality of Strassburg, and it is now being adopted as a pattern throughout central Europe.

THE first time that the Cinematograph was pressed into the service of medicine was, we believe, at the last Berlin Congress, when the movements of the diaphragm and viscera during respiration, as revealed by the Röntgen rays, were clearly reproduced on the lantern screen. A further use of this method of illustration has been made by Dr. H. Campbell Thomson at the opening meeting of the Middlesex Hospital Medical Society to demonstrate different points in the diagnosis and examination of patients in nervous diseases. Dr. Thomson remarked that the Cinematograph might be utilised considerably in medical education, and pointed out that in connection with bacteriology it had become possible to show the movements of micro-organisms and their behaviour in various circumstances, such as the clumping of typhoid bacilli in the Widal reaction. He then proceeded to illustrate the typical diagnostic symptoms of nervous diseases such as disseminated sclerosis, paralysis agitans, Friedreich's disease and pseudo-hypertrophic paralysis, by representing the movements of patients on the screen.

IN the inaugural address at the first meeting of the Electro-therapeutic Section of the Royal Society of Medicine, Mr. W. Deane Butcher gave a comprehensive survey of the progress in the use of electricity for the diagnosis and treatment of disease. Röntgenography and Röntgen-therapy naturally occupy a prominent position, and one of the most recent and important advances in this direction consists in the ability by improved methods to obtain a knowledge of the condition of the internal organs by direct inspection. By the injection of bismuth the shape and movements of the stomach during digestion have been followed. In diseases of the chest the *x*-rays have already proved of considerable utility, especially in the diagnosis of apical infiltrations and of infiltrations of the glands at the hilus of the lung. In a recent issue we referred to the development of orthodiagraphy for the examination of the heart and the diagnosis of cardiac morbid conditions. The time, indeed, seems not far distant when we shall no longer be compelled to rely upon signs and symptoms to form a more or less supposititous diagnosis, but by direct inspection we shall be able to state definitely that such and such a morbid condition exists, and shall be able to watch the changes that take place in that condition for better or worse. A striking instance of these possibilities is afforded by a case of pneumothorax at Guy's Hospital reported by Dr. J. Fawcett. The patient was a man, aged 22, admitted with pneumothorax of the right side and much dyspnœa. As the condition remained unchanged after 19 days the patient was placed on the couch and examined under the *x*-rays. The lung was seen to be compressed towards the spine. A trocar and cannula were inserted into the pleural sac in the sixth interspace and connected with a partially exhausted bottle. The lung was seen then to expand, and when fully expanded the cannula was withdrawn and the puncture sealed. The lung at once acted perfectly to the respiratory movements and the man was discharged well twelve days later.

ONE of the most recently-discovered powers of the electric current and one that especially appeals to the imagination is that of inhibiting the nerve centres and producing temporary unconsciousness after the manner of an anæsthetic. This discovery we owe to the research work of Professor Leduc. The current used is an intermittent one of low tension and unidirectional. The impulses are at the rate of 100 per second and the duration of each one-thousandth of a second. This form of current is produced by a special commutator, and has been found to be the most suitable after a long series of experiments. The current is applied to the head by electrodes placed anteroposteriorly, and it is turned on gradually. As in chloroform narcosis, there is a stage of excitement followed by inhibition and loss of consciousness. The animal shows no sign of pain and reacts to no stimulus. Any operation can be performed. On cutting off the current the animal awakens instantaneously. There are no after-effects, and the animal shows no signs of fright, suffering, or fatigue. So far Professor Leduc himself is the only human being who has been submitted to the current in this way, and with him full narcosis was not allowed to develop. As Mr. Butcher suggests, this research opens up a magnificent and far-reaching vista. By suitable modifications we may be able to produce nerve-inhibitions of all kinds, not only general but local, and the production of electric narcosis may not only take the place of other forms of anæsthesia, but may offer a remedy for conditions of nerve-exhaustion, neurasthenia, and the like.

